# What the Hiccough Is

**Published:** 1893-11

**Authors:** 


ARTICLE VII.

### WHAT THE HICCOUGH IS.

Mr. Wm. H. Veale, who died from hiccoughs, recalls several similar instances, though death from this affection is rare. Ordinarily hiccoughing is a very trifling matter and is the subject of as many jokes as yawning, and serious results from either are about equal in number, and so very rare as to be considered quite wonderful. Sometimes a man breaks his jaw by yawning too hard: that is an occurrence as seldom as death from hiccoughing.

The case of Mr. Veale and its fatal termination has attracted a great deal of attention all over the country, and his wife and physicians have received hundreds of letters, each with suggestions as to treatment, and remedies that were "guaranteed to cure," and the interest in the disease has obtained so great an ascendancy that *The American* requested a leading physician to prepare a treatise upon it.

"Hiccoughing," he says, "or, as it is technically called, 'singuitus,' is a noise made by the sudden and involuntary contraction of the diaphragm, and the simultaneous contraction of the glottis, which arrests the air in the trachea. This clonic spasm of the diaphragm is owing to a reflex action, which is brought about by various conditions. There are two classes of hiccoughs. It may be a symptom of itself; that is, a mere exaggeration of the hiccough of healthy persons, produced by sudden chilling of the stomach by irritant gases and pungent articles. This becomes exaggerated and less repressible than natural hiccoughs by several poisonous agencies, as, for instance, malaria, grip, hay fever, intermittent fever and other blood poisonings. It is different with hiccough due to organic disease in that most important part of the brain, near its junction with the spinal cord. Here the centers for the heart's action, for breathing, stomach and diaphragm movements, including vomiting and hiccoughing, are located. A hemorrhage in this regard no larger than a millet seed can stop the heart's action. A little further up the reactive inflammation around it may cause continuous and distressing vomiting and hiccough, or both, or either combined with paralysis of swallowing.

### A SYMPTOM OF HYSTERIA.

Hiccough is in its mild form a very frequent condition which soon passes off; but in many cases it increases to a persistent, obstinate and very troublesome affection, which may last for weeks or months. It sometimes comes on after mental excitement, and is a not very rare symptom of hysteria. It is also a symptom of other morbid conditions, especially gangrene. Persistent hiccough may also be excited reflexly in affections of the stomach, intestines, peritoneum, etc. In some cases, the hiccough depends upon a direct lesion of the phrenic nerve. Hiccough has also been seen in cerebral apoplexy and also in chronic myelitis, extending to the cervical cord. In these cases the cause, of course, is the serious affection, the hiccough being only a result.

### METHODS OF TREATMENT.

In the milder cases, hiccough soon passes off without special treatment. Holding the breath, pressure on the closed glottis, blows on the back, taking draughts of water, etc., are proceedings generally known to the laity and often used with more or less success to suppress hiccough. In more severe cases, narcotics are tried, as opium, cannabis indica, or inhalations of chloroform. The foradic brush to the region of the diaphragm, or the direct action of electricity on the phranic nerve is sometimes of advantage. In hysterical hiccough, very rapid results may be obtained in this way, or sometimes by one of the different nervous, valerian zinc, atropine or Fowler's solution. Nitro-glycerine has been used with good effect, and camphor has been tried advantageously.

### USEFUL, BUT DANGEROUS.

Nitrite of amyl has also been employed with great success in stopping this troublesome malady. It is generally administered by inhalation, for it will not answer with anything like the same certainty if given by the stomach. It should be borne in mind that it affects some persons more than others, one individual being able to inhale five or ten drops from a handkerchief or to breathe fumes from a bottle held close to the nose, while a whiff from the bottle held at a distance will affect another with great giddiness, much mental confusion and general weakness. Patients may become habituated to it, however, so that after awhile it must be inhaled several times before it affords relief. Nitrate of amyl is considered a powerful and even dangerous remedy, requiring to be watched with great care and to be given in a definite quantity. Mustard has been employed with advantage, and cases of obstinate hiccough are reported which have been immediately cured by drinking an infusion made with a teaspoonful of mustard steeped in four ounces of boiling water for twenty minutes and then strained.

Persons of a highly nervous temperament seem more prone to this affliction than those of a more sanguine disposition, and sometimes forgetfulness of self has been sufficient to stop the hiccough.—*Baltimore American.*

